# The Relationship Between the American Society of Anesthesiologists Physical Status Classification and Patient Outcomes: A Scoping Review Protocol

**DOI:** 10.1111/aas.70221

**Published:** 2026-03-14

**Authors:** Luan Bicalho Costa, Yuri Trivelato Desideri, Ana Carolina Malacco Oliveira, Giovanna Fabris, João Vitor Ferreira Domingos, Ann Merete Møller

**Affiliations:** ^1^ Faculty of Medicine (FAMED) Federal University of Uberlândia Uberlândia Brazil; ^2^ Department of Anaesthesiology, Herlev Hospital University of Copenhagen Copenhagen Denmark

**Keywords:** American Society of Anesthesiologists Physical Status, perioperative outcomes, protocol, risk stratification, scoping review

## Abstract

**Background:**

The American Society of Anesthesiologists Physical Status (ASA‐PS) classification system is ubiquitous in perioperative medicine and research as a tool for preoperative patient risk stratification. Despite widespread clinical adoption as a predictor of perioperative outcomes, the ASA‐PS system is inherently subjective, leading to considerable inter‐rater variability. A comprehensive mapping of the literature examining the relationship between ASA‐PS scores and patient outcomes is lacking.

**Objectives:**

To systematically map the extent, range, and nature of peer‐reviewed literature examining the relationship between the ASA‐PS classification and patient outcomes, and to identify key characteristics, themes, and knowledge gaps in this evidence base.

**Methods:**

This scoping review will be conducted according to the Joanna Briggs Institute (JBI) methodological framework and reported using the Preferred Reporting Items for Systematic Review and Meta‐Analysis extension for Scoping Reviews (PRISMA‐ScR). The Population‐Concept‐Context (PCC) framework will guide eligibility assessment. A comprehensive search will be conducted across PubMed, EMBASE, Scopus, LILACS, and the Cochrane Central Register of Controlled Trials, with no language or date restrictions. Study selection will be performed independently and in duplicate by two reviewers in two stages (title/abstract screening, full‐text review). If any discordance appears, a third reviewer verdict will be requested. Data will be extracted using a structured charting form and synthesized narratively.

**Context:**

Any healthcare setting where an ASA‐PS score is assigned prior to a procedure (inpatient hospital, ambulatory surgery center, outpatient clinic). Primary research designs, including randomized controlled trials, observational studies (cohort, case–control, cross‐sectional, descriptive), and case reports will be eligible; review articles, editorials, letters to the editor, and commentaries will be excluded.

**Search Strategy:**

The search will employ controlled vocabulary (MeSH terms) and free‐text keywords including: “ASA score,” “ASA Physical Status Classification System,” “American Society of Anesthesiologists,” in combination with outcome‐related terms. Supplementary hand searching of reference lists and Google Scholar will be performed.

**Data Extraction:**

Study characteristics (author, year, country, journal, design), population characteristics (sample size, age, comorbidity), context (clinical setting, specialty, procedure type, urgency), ASA score details, and outcome details (including statistical methods used to derive associations) will be extracted. A preliminary data charting form is provided in Appendix B.

**Synthesis:**

Narrative synthesis supported by descriptive statistics will map study characteristics, outcome categories, clinical contexts, study designs, and temporal and geographical distribution of research. No formal quality appraisal will be conducted.

**Ethics and Registration:**

Ethics committee approval is not required for this protocol‐based scoping review.

## 1 Introduction

### 1.1 Background: The ASA Physical Status Classification System

The American Society of Anesthesiologists Physical Status (ASA‐PS) classification system is a foundational tool in modern perioperative medicine, providing a standardized method for assessing and categorizing a patient's physiological status before surgery [1]. Its origins trace back to 1940–1941, initially conceived as a system for the collection and tabulation of statistical data in anesthesia. The primary goal was to establish a common terminology that would allow for meaningful statistical comparisons of morbidity and mortality by relating surgical outcomes to the patient's preoperative condition [1, 2]. From its inception, the system was explicitly designed to describe the patient's preoperative state only, not to function as a direct predictor of operative risk for an individual.

The initial six‐point scale proposed by Saklad and colleagues was refined over the decades, and the version adopted by the American Society of Anesthesiologists in 1963 forms the basis of the modern classification. The current system comprises six classes, ranging from ASA I (a normal healthy patient) to ASA VI (a declared brain‐dead patient whose organs are being removed for donor purposes). An “E” modifier is appended to the classification to denote an emergency procedure, defined as a situation where a delay in treatment would significantly increase the threat to the patient's life or a body part. The six classes are defined as follows:

- *ASA I*: A normal healthy patient.
- *ASA II*: A patient with mild systemic disease.
- *ASA III*: A patient with severe systemic disease.
- *ASA IV*: A patient with severe systemic disease that is a constant threat to life.
- *ASA V*: A moribund patient who is not expected to survive without the operation.
- *ASA VI*: A declared brain‐dead patient whose organs are being removed for donor purposes.

### 1.2 The ASA Score as a De Facto Predictor of Patient Outcomes

The ASA‐PS score has evolved to become one of the most widely used instruments in clinical practice for communicating a patient's overall health status and, by extension, for forecasting perioperative risk [1, 3]. Its simplicity and universality have led to its integration into routine preoperative assessments worldwide. It is frequently used to inform clinical decision‐making, guide the allocation of healthcare resources (such as postoperative intensive care unit [ICU] beds), and, in some healthcare systems, to adjust billing and reimbursement [1].

This widespread adoption has been fueled by an extensive body of literature that has consistently demonstrated a strong correlation between higher ASA scores (typically ASA III and above) and a broad spectrum of adverse patient outcomes [1, 2, 3].

### 1.3 Rationale for a Scoping Review: Mapping a Broad and Heterogeneous Evidence Base

The ubiquitous application of the ASA score as a risk stratification tool is juxtaposed with its most significant and well‐documented limitation: its inherent subjectivity [1]. The definitions for each class are qualitative (e.g., “mild” vs. “severe” systemic disease), which can lead to considerable inter‐rater variability among clinicians [1]. Studies have shown that different anesthesiologists may assign different ASA scores to the same patient, a discrepancy that has profound implications for both clinical care and the interpretation of research findings [1, 3].

This fundamental tension—between the score's widespread use as a risk predictor and its subjective nature—has given rise to a vast, complex, and highly heterogeneous body of literature. Research examining the ASA score's relationship with patient outcomes spans nearly every surgical and medical specialty, involves diverse patient populations, measures a multitude of different outcomes, and employs a wide variety of study designs. This creates a research landscape that is exceptionally broad and multifaceted.

In this context, a scoping review is the most suitable methodology for this inquiry. A scoping review functions as a form of knowledge synthesis that systematically maps the existing evidence, identifies its key characteristics, and highlights gaps in the research, all without formally appraising the methodological quality of the individual studies included [4]. It is, in essence, a cartographic exercise designed to chart the contours of a broad field of research.

### 1.4 Primary Objectives and Research Questions

The primary objective of this scoping review is to systematically map the extent, range, and nature of the peer‐reviewed literature that examines the relationship between the ASA Physical Status classification and patient outcomes. To achieve this objective, this review will address the following specific research questions:

- What is the volume, chronological distribution, and geographical origin of the literature on this topic?
- What categories of patient outcomes (e.g., mortality, morbidity, healthcare utilization, patient‐reported outcomes) have been examined in relation to the ASA score?
- In which medical contexts (e.g., surgical specialties, medical disciplines, emergency vs. elective settings, specific patient populations) has this relationship been investigated?
- What are the characteristics of the available evidence, including the predominant study designs, data sources, and other methodological features employed?
- What main themes, concepts, and reported limitations of the ASA score emerge from the literature?
- What are the key knowledge gaps in the existing literature that could inform a future research agenda?

## 2 Methods

### 2.1 Methodological Approach and Reporting Standards

This protocol has been developed in accordance with the Preferred Reporting Items for Systematic Review and Meta‐Analysis Protocols (PRISMA‐P) guidelines [5]. The subsequent scoping review will be conducted and reported in adherence to the PRISMA extension for Scoping Reviews (PRISMA‐ScR) checklist [6] (Appendix C) and the methodological framework for scoping reviews developed by the Joanna Briggs Institute (JBI) [4]. Assistance in editing portions of this protocol was obtained from an AI language model (Perplexity, powered by GPT‐5.1, https://www.perplexity.ai/page/openai‐launches‐gpt‐5‐1‐with‐p‐4xtQ5cBdQzCP1tBjOOrsUA; Perplexity AI Inc., San Francisco, CA, USA). The authors have reviewed and take full responsibility for all content.

#### 2.1.1 Status of the Review

This is a protocol manuscript submitted prior to commencing the formal scoping review. The search strategy has been developed and refined iteratively in collaboration with a medical information specialist to optimize retrieval and align with the review objectives. A preliminary exploratory search was conducted by the main author to assess the scope and characteristics of the existing literature and inform protocol development. No formal systematic searching, screening, or data extraction has been initiated as of the date of submission (February 2026).

### 2.2 Eligibility Criteria: Population, Concept, and Context Framework

The selection of studies for this review will be guided by the Population, Concept, and Context (PCC) framework, as recommended by the JBI for scoping reviews. The specific inclusion and exclusion criteria are detailed in Table 1.

**TABLE 1:** Eligibility criteria based on the PCC framework.

PCC component	Inclusion criteria	Exclusion criteria
Population	Patients with ASA‐PS score assigned.	Animal studies.
Concept	Studies examining the relationship between the ASA‐PS score and at least one patient outcome. Patient outcomes include mortality, morbidity, postoperative complications, length of stay, ICU admission, readmission, costs, and patient‐reported outcomes.	Studies that use the ASA score but do not report on its relationship with a patient outcome. Studies on the development or validation of the ASA score itself without linking it to outcomes. Studies that use ASA score to build a new scoring system or as training data to predict outcomes.
Context	Any healthcare setting (hospital, ambulatory surgery center, clinic). No restrictions on geographical location, publication date, or language of publication. Primary research studies (RCTs, cohort, case–control, cross‐sectional studies).	Non‐healthcare settings. Studies with full text unavailable. Review articles, editorials, letters to the editor, and commentaries.

*Population*: The population of interest includes all studies involving patients who have been assigned an ASA‐PS score in a healthcare context. Studies that focus on animals will be excluded.

*Concept*: The core concept is the examination of the relationship between the ASA‐PS score, acting as a predictor or stratifying variable, and any reported patient postoperative outcome. The term “patient outcome” will be interpreted broadly to encompass:

- Mortality (all‐cause, procedure‐specific, in‐hospital, 30‐day);
- Morbidity (any postoperative medical or surgical complication):
- Measures of healthcare utilization (length of stay, ICU admission, readmission rates, costs);
- Patient‐reported outcomes (quality of life, functional status, satisfaction)

*Context*: The context for this review is any healthcare setting where an ASA score is assigned prior to a procedure. This includes inpatient hospitals, ambulatory or day‐surgery centers, and outpatient clinics. No restrictions will be placed on the geographical location of the study, the type of healthcare system, or the date of publication. All research study designs will be considered eligible for inclusion, including randomized controlled trials, observational studies (cohort, case–control, cross‐sectional, descriptive studies), and case reports. Review articles, editorials, letters to the editor, and commentaries will be excluded.

### 2.3 Information Sources and Search Strategy

A comprehensive and systematic search strategy will be designed and executed in collaboration with an experienced medical information specialist. The search will be conducted across several major electronic databases, including:

- PubMed (MEDLINE);
- EMBASE;
- Scopus;
- LILACS (Latin American and Caribbean Health Sciences Literature);
- Cochrane Central Register of Controlled Trials (CENTRAL).

To maximize the sensitivity of the search, no language or date filters will be applied during the initial database search. The search strategy employs controlled vocabulary (MeSH terms and EMTREE terms) combined with free‐text keywords to ensure comprehensive retrieval. A preliminary example of the search strategy is provided in Appendix A.

In addition to the electronic search, hand‐searching will be employed, wherein the reference lists of all included studies and relevant review articles will be scanned to identify any potentially eligible studies that were not captured by the initial search. A free search in Google Scholar will be conducted with the intention of identifying any additional potentially eligible studies. Grey literature will be considered selectively to minimize publication bias while maintaining feasibility.

### 2.4 Study Selection Process

All records identified through the database searches will be imported into Rayyan (a web and mobile app for systematic reviews) to facilitate the screening process. Duplicate records will be removed both automatically by the software and through manual verification.

The study selection will be conducted in a two‐stage process by two independent reviewers, a standard procedure to ensure rigor and minimize bias:

*Stage 1—Title and Abstract Screening*: Two reviewers will independently screen the titles and abstracts of all retrieved records in duplicate against the predefined eligibility criteria. Any record that clearly does not meet the criteria will be excluded at this stage.

*Stage 2—Full‐Text Review*: The full‐text articles for all records deemed potentially relevant during Stage 1 will be retrieved. The same two reviewers will then independently and in duplicate assess these full‐text articles against the eligibility criteria to make a final inclusion decision.

Any disagreements between the reviewers at either stage of the screening process will be resolved through discussion and consensus. If a consensus cannot be reached, a third, senior reviewer will be consulted to arbitrate a final decision. The entire selection process will be documented, and the reasons for excluding studies at the full‐text stage will be recorded. A PRISMA flow diagram will be used in the final scoping review to transparently report the flow of studies through the review process.

### 2.5 Data Charting and Extraction

A structured data charting form will be developed by the research team to guide the extraction of relevant information from the included studies. This form will be refined based on pilot testing to ensure clarity, comprehensiveness, and consistent application by all members of the review team. A preliminary version of the data charting form is provided in Appendix B (Table B1).

Data from each included study will be extracted by one reviewer and subsequently verified for accuracy and completeness by a second reviewer. The following data items will be charted:

**Study Characteristics:**
- First author, year of publication, country of origin, journal, and study design.

**Population Characteristics:**
- Total sample size, patient age (mean, median, or range), comorbidity burden.

**Context:**
- Clinical setting (e.g., academic hospital, community center);
- Relevant medical or surgical specialty:
- Details of procedures studied:
- Urgency of procedure (e.g., elective vs. emergency).

**ASA Score Details:**
- Information on how the ASA score was determined and reported in the study, including timing of assignment (if available):
- Distribution of patients across ASA classes.

**Outcome Details:**
- Specific patient outcomes measured:
- Definitions used for each outcome:
- Reported associations between ASA score and outcomes (e.g., odds ratios, hazard ratios, *p*‐values, or narrative descriptions);
- Statistical methods and models used to derive reported associations where reported.

**Key Findings**
- A summary of the study's main findings regarding the reported relationship between the ASA score and measured patient outcomes

### 2.6 Data Synthesis and Analysis

In line with the standard methodology for scoping reviews, the extracted data will be synthesized without a formal appraisal of the quality or risk of bias of the included studies. The synthesis will be primarily a narrative summary of the findings, structured around the review's research questions.

This narrative will be supported by descriptive statistics (e.g., frequencies, percentages, rates) and presented using tables and graphical formats (e.g., bar charts, scatter plots, bubble charts). This approach will allow for a clear and comprehensive mapping of the evidence, for example, by using charts to visualize:

- The distribution of research across different clinical specialties and surgical subspecialties;
- The types of patient outcomes investigated;
- The predominant study designs employed;
- The chronological distribution of publications;
- The geographical distribution of included studies;
- Key themes and findings regarding the ASA score's predictive value and limitations.

The narrative synthesis will specifically address the six research questions outlined in Section 1.4 and identify recurring themes, reported limitations, and identified knowledge gaps.

### 2.7 Ethics and Dissemination

#### 2.7.1 Ethics Approval

As this scoping review is based on published, aggregate data from secondary sources, ethics committee approval is not required. However, the findings will be reported transparently and in adherence to established reporting standards.

#### 2.7.2 Dissemination

The findings of this scoping review will be disseminated through:

- Publication in a peer‐reviewed journal;
- Presentations at relevant conferences (e.g., Scandinavian Society of Anaesthesiologists annual meeting).

## 3 Anticipated Contributions and Expected Utility

### 3.1 Expected Utility and Implications for Research and Practice

This scoping review is poised to deliver a comprehensive, systematic map of the vast and influential body of literature that examines the relationship between the ASA score and patient outcomes. The findings from this review will hold significant value for a diverse range of stakeholders in the healthcare ecosystem.

For clinicians and medical educators, the review will provide a structured and accessible overview of the evidence base that illustrates the relationship between patient outcomes and the ASA score. This can be used to enhance preoperative patient counseling, improve shared decision‐making, and facilitate more nuanced discussions about perioperative risk.

For the research community, the systematic charting of the existing literature will be instrumental in identifying critical knowledge gaps. For example, the review may reveal:

- A relative scarcity of research on the ASA score's association with patient‐reported outcomes (e.g., long‐term quality of life or functional status) compared to traditional outcomes like mortality;
- Specific clinical populations or surgical specialties where this relationship has been under‐investigated;
- Geographic or methodological gaps in the literature.

These identified gaps will provide a clear, evidence‐based roadmap to guide future primary research and prioritize areas for further investigation.

For healthcare systems and policymakers, understanding the breadth and limitations of the evidence base can inform risk stratification policies, resource allocation decisions, and quality improvement initiatives.

A significant contribution of this review will be to systematically document the evidence‐practice paradox at the core of the ASA score's modern use. By juxtaposing the score's widespread acceptance in research and clinical practice with its well‐documented methodological flaws (particularly subjectivity and inter‐rater variability), this review can stimulate a more critical and informed discourse regarding appropriate applications and limitations of the ASA‐PS classification system.

### 3.2 Strengths and Limitations of the Review Methodology

**Strengths:**
- The primary strength of this scoping review is its foundation in a rigorous, transparent, and reproducible methodology;
- The protocol is designed in accordance with established international standards, including the PRISMA‐ScR and JBI guidelines;
- The development of a comprehensive, multi‐database search strategy in collaboration with an information specialist is designed to ensure that the full breadth of the relevant literature is captured;
- The two‐stage, dual‐reviewer study selection process minimizes selection bias;
- Structured data extraction and charting will ensure comprehensive documentation of study characteristics and findings.

**Limitations:**
- The principal limitation is inherent to the scoping review methodology itself: the absence of a formal risk‐of‐bias assessment or critical appraisal of the included studies;
- As a result, this review will describe the state of the evidence but will not make summative judgments about the methodological quality of that evidence or the true magnitude and validity of the reported associations between the ASA score and patient outcomes;
- The objective is to map the existing literature, not to weigh the evidence or synthesize a definitive answer regarding the predictive accuracy of the ASA‐PS classification system;
- Heterogeneity in study designs, outcome definitions, and populations may limit the ability to draw comparative conclusions across studies;
- Language and availability restrictions may result in some relevant literature being excluded.

### 3.3 Conclusion

The American Society of Anesthesiologists Physical Status classification system is a cornerstone of preoperative patient assessment and a ubiquitous variable in perioperative research. This scoping review will provide a comprehensive and structured overview of the extensive literature that has investigated its relationship with a wide array of patient outcomes. The findings will be instrumental in identifying key characteristics and gaps in the current body of knowledge. This, in turn, will inform the direction of future research, ultimately contributing to the ongoing effort to refine perioperative risk stratification and improve the safety and quality of patient care.

## Author Contributions

**Luan Bicalho Costa:** conceptualization, methodology, investigation, writing – original draft, project administration. **Yuri Trivelato Desideri:** investigation, writing – review and editing. **Ana Carolina Malacco Oliveira:** investigation, writing – review and editing. **Giovanna Fabris:** investigation, writing – review and editing. **João Vitor Ferreira Domingos:** investigation, writing – review and editing. **Ann Merete Møller:** conceptualization, methodology, supervision, writing – review and editing.

## Funding

The authors have nothing to report.

## Conflicts of Interest

The authors declare no conflicts of interest.

## Data Availability

This is a protocol for a scoping review. Data sharing is not applicable at this stage. Upon completion, the data generated and analyzed during this review will be available from the corresponding author upon reasonable request.

## Appendix A Preliminary Search Strategy

The following search terms will be adapted for each database using appropriate controlled vocabulary and syntax (Table A1):

**TABLE A1:** Example PubMed search strategy.

Search	Actions	Details	Query	Results
#3			Search: ***#2 AND #1***	1,142
#2			Search: ***“Postoperative Outcome*” OR “Patient Outcome Assessment*” OR “Patient Outcome*”***	149,730
#1			Search: ***“ASA score*” OR “ASA Physical Status Classification System” OR “ASA Classification” OR “ASA Status” OR “ASA Physical Status” OR “American Society of Anesthesiologists score” OR “American Society of Anesthesiologists Physical Status Classification System” OR “American Society of Anesthesiologists Classification” OR “American Society of Anesthesiologists Status” OR “American Society of Anesthesiologists Physical Status”***	16,712

*Note:* The search strategy will be refined iteratively and adapted using database‐specific controlled vocabulary and syntax.

## Appendix B Preliminary Data Charting Form (Table B1)

**TABLE B1:** Data extraction form.

Data item	Description
Study identifiers	
First author	Last name of the first author
Year of publication	Year the study was published
Journal	Name of the journal
Country of origin	Country where the study was conducted
Study characteristics	
Study aim/objective	The stated purpose of the study
Study design	For example, RCT, prospective cohort, retrospective cohort, case–control, cross‐sectional
Population characteristics	
Sample size	Total number of patients included in the analysis
Age	Mean, median, or range of patient ages
Comorbidity burden	Assessment of patient comorbidities
Context	
Clinical setting	For example, inpatient, ambulatory, single‐center, multi‐center
Medical/surgical specialty	For example, general surgery, orthopedics, cardiology, obstetrics, neurosurgery
Procedure type	Specific procedures studied (e.g., total hip arthroplasty, coronary artery bypass graft)
Urgency	For example, elective, emergency, or mixed
ASA score details	
Method of assignment	How the ASA score was assigned and by whom (if reported)
ASA score distribution	Breakdown of patients by ASA class (*n*, %)
Outcome details	
Outcomes measured	Name of primary and secondary outcomes (e.g., 30‐day mortality, surgical site infection)
Outcome definition	How each outcome was defined by the authors
Reported association	Summary of findings (e.g., ORs, HRs, *p*‐values, or narrative description)
Statistical methods/models	Methods used to derive reported associations, including type of model (e.g., univariable or multivariable logistic regression, Cox regression, linear regression, propensity score methods) and key covariates included in adjusted analyses, where reported
Key findings and limitations	
Key findings	Summary of the main findings regarding the ASA‐outcome relationship
Study limitations	Author discussion of study limitations, including ASA score limitations
Notes	Any other relevant information

## Appendix C

This protocol was developed in accordance with the PRISMA extension for Scoping Reviews (PRISMA‐ScR) [6]. Items that apply only to completed scoping reviews (e.g., results and discussion of findings) are marked as not applicable (N/A) at the protocol stage (see Table C1).

**TABLE C1:** PRISMA‐ScR checklist for the protocol.

Section/item	PRISMA‐ScR item	Location in protocol/status
Title (Item 1)	Identify the report as a scoping review protocol.	Title page; Abstract
Abstract (Item 2)	Provide a structured summary including background, objectives, methods (eligibility criteria, information sources, charting methods), and planned synthesis.	Abstract
Rationale (Item 3)	Describe the rationale for the review in the context of what is already known.	Sections 1.1, 1.3
Objectives (Item 4)	Provide an explicit statement of the questions and objectives being addressed with reference to key elements (population, concept, context).	Section 1.4
Protocol and registration (Item 5)	Indicate whether a review protocol exists, where it can be accessed, and whether it is registered.	Section 2.1.1
Eligibility criteria (Item 6)	Specify characteristics of the sources of evidence used as eligibility criteria (e.g., PCC elements, type of evidence sources, publication status, language).	Section 2.2; Table 1
Information sources (Item 7)	Describe all intended information sources (e.g., databases, contact with authors, trial registers, grey literature) and planned coverage dates.	Section 2.3
Search (Item 8)	Present a draft search strategy for at least one database, including planned limits, to be used for the review.	Section 2.3; Appendix A
Selection of sources of evidence (Item 9)	State the process for selecting sources of evidence (screening and eligibility) to be included in the scoping review.	Section 2.4
Data charting process (Item 10)	Describe the planned methods for charting data from included studies, including whether charting will be done independently and in duplicate, and how disagreements will be resolved.	Section 2.5
Data items (Item 11)	List and define all variables for which data will be sought (e.g., PCC elements, outcomes, study characteristics).	Section 2.5; Appendix B (Table B1)
Critical appraisal of individual sources of evidence (Item 12)	If planned, describe methods for critical appraisal of individual sources of evidence.	Not planned; stated in Section 2.6 (no formal quality appraisal).
Synthesis of results (Item 13)	Describe the planned methods of handling and summarizing the data that will be charted.	Section 2.6
Results (Items 14–18)	Characteristics of sources of evidence, results of individual sources, synthesis of results.	N/A at protocol stage.
Discussion (Items 19–21)	Summary of evidence, limitations, and conclusions for the completed review.	N/A at protocol stage (anticipated contributions detailed in Section 3).
Funding (Item 22)	Describe sources of funding for the review and the role of the funders.	Funding statement (end of document).
